# The Role of the PI3K/Akt/mTOR Axis in Head and Neck Squamous Cell Carcinoma

**DOI:** 10.3390/biomedicines12071610

**Published:** 2024-07-19

**Authors:** Qian Jiang, Jingyi Xiao, Yao-Ching Hsieh, Neha Love Kumar, Lei Han, Yuntao Zou, Huang Li

**Affiliations:** 1 Nanjing Stomatological Hospital, Affiliated Hospital of Medical School, Institute of Stomatology, Nanjing University, Nanjing 210093, China; qian.jiang@ucsf.edu (Q.J.);; 2 International Dentist Pathway, University of California, San Francisco, CA 94158, USA; 3 Division of Hospital Medicine, University of California, San Francisco, CA 94158, USA

**Keywords:** HNSCC, PAM signaling pathway, inhibitors, resistance

## Abstract

Head and neck squamous cell carcinoma (HNSCC) is one of the most common malignancies globally, representing a significant public health problem with a poor prognosis. The development of efficient therapeutic strategies for HNSCC prevention and treatment is urgently needed. The PI3K/AKT/mTOR (PAM) signaling pathway is a highly conserved transduction network in eukaryotic cells that promotes cell survival, growth, and cycle progression. Dysfunction in components of this pathway, such as hyperactivity of PI3K, loss of PTEN function, and gain-of-function mutations in *AKT*, are well-known drivers of treatment resistance and disease progression in cancer. In this review, we discuss the major mutations and dysregulations in the PAM signaling pathway in HNSCC. We highlight the results of clinical trials involving inhibitors targeting the PAM signaling pathway as a strategy for treating HNSCC. Additionally, we examine the primary mechanisms of resistance to drugs targeting the PAM pathway and potential therapeutic strategies.

## 1. Introduction

Head and neck squamous cell carcinoma (HNSCC), arising in the oral cavity, larynx, and pharynx, is one of the most common malignancies, ranked sixth worldwide, and affects 600,000 patients annually [1]. HNSCC has a poor prognosis, with only 40–50% surviving more than 5 years [2]. Smoking and alcohol consumption are recognized risk factors for the development of HNC. Meanwhile, viral infection is considered a risk factor associated with the development of HNC subgroups. Infection with specific types of human papillomavirus (HPV) is highly correlated with oropharyngeal cancer, with HPV16 being the most common genotype [3,4]. Currently, surgery, radiation, and chemotherapy are the main therapies for HNSCC. However, these nonselective treatments are not tolerated well with severe systemic complications. While surgery or radiation therapy can often lead to a cure for most patients with early-stage HNSCC, those facing aggressive forms of the disease or those in advanced stages, which account for approximately two-thirds of new diagnoses, tend to have a higher likelihood of recurrence, with a 50% overall survival rate over five years [5]. There are only three targeted drugs approved by the FDA: the epidermal growth factor receptor (EGFR) antibody cetuximab and the programmed death receptor-1 (PD-1) antibodies pembrolizumab and nivolumab [6,7,8]. Cetuximab or pembrolizumab may cause life-threatening thromboembolism [9]. The risk of venous or pulmonary embolism increases approximately 1.5 times with cetuximab or pembrolizumab treatment [9]. Cisplatin is the agent suggested for HNSCC to use in the adjuvant setting [10], which can be associated with significant side effects including kidney disease, blood or bone marrow problems. A new, more targeted therapy is needed to improve the current treatment plan for HNSCC. Understanding the gene alterations involved in HNSCC formation and progression would help develop novel precision therapeutic options. Precision medicine focuses on tailoring treatment plans for patients. One approach for precision medicine is to predict the behavior of a type of tumor based on big omics data analysis, which involves characterizing each tumor’s molecular and genetic features through multiple omics analyses of tumor samples [11]. Institutions worldwide contribute to the sharing of big omics data on tumors through international projects. The other approach is to develop personalized preclinical platforms using in vitro and in vivo techniques to test the behavior and drug sensitivity of samples derived from patients [11]. Deep sequencing of the genomic landscape of HNSCC reveals the diversity of genetic alterations in this malignancy. Although many specific molecules are altered in each tumor, they all involve only a few driving signaling pathways. Among these, the PAM pathway is most frequently activated, playing a central role in cancer development and progression [12]. In turn, targeting the PAM pathway may represent a precision treatment approach for HNSCC. It should be noted that dysregulation of the PAM pathway is common in both HPV-positive and -negative HNSCC [13]. Viral oncogenes such as HPV E6 and E7 can release regulation of EGFR/PI3K/Akt/mTOR and increase *PIK3CA* gene mutations [14]. Targeted therapy targeting the PAM pathway is a promising treatment strategy for HPV-positive and HPV-negative HNSCC.

Phosphoinositide 3-kinases (PI3Ks) are a family of enzymes that play crucial roles in various cellular functions, including cell growth, proliferation, and differentiation, which in turn are integral to cancer development and progression. It has been reported that the most mutated and amplified oncogene in human cancers, including HNSCC, is PI3K catalytic subunit alpha isoform (*PIK3CA*), the gene that programs for the p110a isoform of PI3K [15]. PI3K is supposed to be regulated by the tumor suppressor phosphatase and tensin homolog (PTEN). So an inactivating mutation or loss of *PTEN* can induce the formation of cancer by releasing the suppression of the PI3K pathway [16]. It has also been reported that other effectors like protein kinase B (Akt) and mammalian target of rapamycin (mTOR), which are downstream of PI3K, also play important roles in carcinogenesis. Together, the members of the PI3K/Akt/mTOR axis function as a group to interact with the regulation of several other signaling molecules in HNSCC [17]. The PAM pathway is upregulated in over 90% of HNSCC cases [18]. Upregulated PAM signaling would worsen radiotherapy resistance and cytostatic drug resistance [8]. As a result, developing pharmacologic inhibitors targeting the PAM axis has potential benefits for HNSCC patients, which have later been reported in several initial preclinical and clinical studies.

### 1.1. The PAM Signaling Pathway Composition and Function

**Figure 1:**
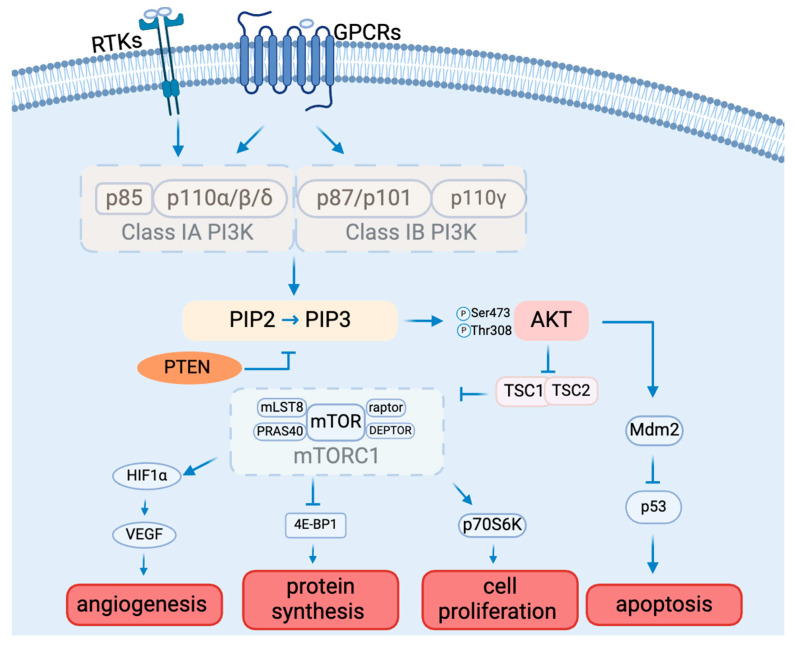
The PAM signaling pathway composition and function.

The PAM signaling cascade is critical to cell survival, growth, proliferation, angiogenesis, transcription, and apoptosis [19] (Figure 1). Various molecules, including glucose, insulin, and many cytokines and growth factors, can initiate PAM signaling [20].

### 1.2. PI3Ks

There are three classes of PI3Ks: class I, class II, and class III, classified by structure and substrate preference [21]. Class I PI3Ks can be activated directly by cell surface receptors, which are best studied among all classes. Class I PI3Ks are divided into subclasses IA and IB based on their mode of activation [21]. The difference is that one can be activated by RTKs, G protein-coupled receptors, and the small G protein RAS (Class IA), and the other one can only be activated by G-protein-coupled receptors (Class 1B) [22]. Class IA PI3Ks are heterodimers made up of a p110 catalytic subunit (p110α, p110β, or p110δ) and a p85 regulatory subunit (p85α, p85β, p85γ, p55α, or p50α). Class IB PI3K consists of a p110γ catalytic subunit and a p101 or p87 regulatory subunit. [23]. P110α and p110β are widely expressed in multiple tissues. In contrast, p110δ and p110γ are mainly found in leukocytes [24,25]. Cell surface receptor activation results in the tyrosine phosphorylation of cell surface receptors. The p85 regulatory subunit of class IA PI3Ks binds directly to tyrosine receptors on cell membranes. The activated PI3K consequently catalyzes the conversion of PIP2 to PIP3 [26]. PIP3 functions as a second messenger, controlling numerous downstream signaling pathways [26].

### 1.3. AKT

Akt is the main molecule downstream of the PI3K signaling pathway, and it is a serine/threonine protein kinase with three isoforms, Akt1, Akt2, and Akt3, which are encoded by PKBα, PKBβ, and PKBγ, respectively [27,28]. Akt requires phosphorylation for activation. AKT1 is extensively expressed in numerous tissues. While AKT2 is found mostly in insulin-sensitive tissues and at low levels in other tissues, AKT3 is solely expressed in the brain and testis [29]. The distinct tissue expression patterns of the various AKT subtypes point to their crucial involvement in the preservation of physiological processes in various organs or tissues [30]. The three AKT subtypes have extremely similar three-dimensional structures made up of three distinct functional domains [31]. The N-terminal pleckstrin homology (PH) domain interacts with PI3K and launches recruitment to the plasma membrane, conformational change, and subsequent phosphorylation [32]. The central kinase catalytic domain shares a high degree of homology with the enzymatic activity regions of protein kinase A (PKA) and protein kinase C (PKC) [33]. Moreover, AKT activation requires the phosphorylation of Thr308, which is situated in this domain. For full activation of AKT, Ser473 needs to be in the regulatory region of the C-terminal AKT domain [34,35]. Akt, once activated, engages with numerous downstream targets implicated in fostering cell survival, growth, proliferation, angiogenesis, metabolism, and migration [36,37,38].

### 1.4. mTOR

The mechanistic target of rapamycin (mTOR) is a protein kinase that is a specific target of the natural compound rapamycin. It is also referred to as RAFT1 (rapamycin and FKBP12 target), RAPT 1 (rapamycin target 1), SEP (sirolimus effector protein), or FKBP12-rapamcyin-associated protein (FRAP) [39]. This 289 kDa serine/threonine kinase is a member of the PI3K-related protein kinase (PIKK) family because its C-terminus is highly homologous to the catalytic domain of PI3K [39]. Mammalian target of rapamycin complex 1 (mTORC1) and mammalian target of rapamycin complex 2 (mTORC2) are two physically and functionally different complexes that are formed by mTOR.

mTORC1 mainly regulates cell growth and metabolism [40]. mTORC1 consists of mTOR (mammalian target of rapamycin), raptor (regulatory-associated protein of mTOR), mLST8 (mammalian lethal with SEC13 protein 8), and two negative regulators, PRAS40 (proline-rich AKT substrate 40 kDa) and DEPTOR (DEP domain-containing mTOR-interacting protein), and is inhibited by rapamycin. mTORC2 mainly controls cell proliferation and survival, which is composed of mTOR (mammalian target of rapamycin), Rictor (rapamycin-insensitive companion of mTOR), mLST8 (mammalian lethal with SEC13 protein 8), SIN1 (mammalian stress-activated protein kinase-interacting protein), PRR5 (protein observed with Rictor 1), and Protor2 [39]. mTORC2 can indeed be activated by growth factors such as insulin and IGF-1, ultimately leading to the phosphorylation and activation of Akt by phosphorylating Ser473 as part of the insulin/IGF-1 signaling pathway [41]. As a downstream molecule of AKT, phosphorylated AKT activates mTORC1 by phosphorylating the tuberous sclerosis complex (TSC). In addition to controlling mTOR activity, the AKT/TSC1-TSC2 signaling pathway also controls cell division and growth. mTORC1 activation requires TSC2’s GTPase activity and function of inhibiting the small GTPase Rheb [42]. Once TSC2 is phosphorylated by AKT, it can no longer inhibit mTORC1. The activated mTOR can further induce angiogenesis by activating hypoxia-inducible factor 1 alpha (HIF-1α), which can further activate vascular endothelial growth factor (VEGF), the important factor for angiogenesis [43]. The monoclonal antibody medication bevacizumab targets multiple cancers by inhibiting VEGF [44]. mTOR can also affect protein synthesis by inhibiting factor 4E-binding protein 1 (4E-BP1) [45,46] and cell proliferation by activating ribosomal protein S6 kinase beta-1 (S6K1), also known as p70S6 kinase (p70S6K) [47]. 4E-BP1 and S6K1 may also be involved in the regulation of H1F-1α [43].

### 1.5. Other AKT Target Proteins

The PI3K/AKT signaling pathway adversely regulates the transcription factors known as forkhead box O (FOXO), which are thought to have an inhibitory influence on cell proliferation. By downregulating FOXO and activating AKT and other targets, PI3K signaling controls the growth of cells [27]. 

Glycogen synthase kinase-3 (GSK-3) is another important molecule downstream of AKT, and it is a serine/threonine protease. There are two subtypes of GSK-3: GSK-3beta and GSK-3alpha, with 97% sequence identity in the catalytically active areas of these two kinds. Furthermore, GSK-3beta and GSK-3alpha are widely expressed in cells and organs and share similar biological characteristics [48]. GSK-3beta has been shown in recent research to be able to phosphorylate a wide range of endogenous substrates, including many proteins and transcription factors that are important in metabolism. Thus, GSK-3beta is essential for cell proliferation, development, carcinogenesis, and glucose homeostasis control [49,50,51]. 

Mdm2 is another substrate of AKT. When AKT phosphorylates Mdm2, it leads to the downregulation of p53 [52]. This reduction in p53 levels promotes autophagy. Thus, the activation of the PI3K/AKT signaling pathway and its phosphorylation of downstream Mdm2 may play a key role in the mechanism of autophagy-induced apoptosis [53].

### 1.6. PTEN

Numerous variables regulate the PI3K/AKT/mTOR signal transduction pathway. The opposing process of PIP3 production is catalyzed by the tumor suppressor PTEN, which changes PIP3 into PIP2 [54]. PTEN hinders cell division and disrupts cellular metabolism by downregulating the PI3K/AKT/mTOR pathway, so PTEN activity inhibition triggers AKT and related pathways [55]. By modifying AKT activity, PTEN has a significant impact on glucose homeostasis regulation [56].

## 2. The Roles of the PAM Pathway in Head and Neck Cancer

The PAM pathway is a highly conserved signal transduction network in eukaryotic cells that promotes cell growth, survival, and cycle progression [57]. The malfunction of PAM signal transduction may be a risk factor for the development of cancer [38]. The PAM pathway abnormality is found in about half of malignancies [57]. Furthermore, treatment resistance in cancer is often a result of hyperactivation of the PAM pathway [57,58].

### 2.1. PI3K Mutations in HNSCC

The most often found mutant oncogene throughout tumor lineages is the *PIK3CA* activating mutation, which encodes the p110α catalytic subunit of PI3K. This mutations not only present in HNSCC [59], but also detected in gastric cancer [60], gallbladder cancer [61], and melanoma [62]. Eighty percent of the alterations reported by the HNSCC were missense mutations in *PIK3CA* [63]. The p.(E545K) mutation, which results from a single nucleotide change (c.1633 G > A) and substitutes lysine (K) for the amino acid (AA) glutamic acid (E) at position 545 in the *PIK3CA* gene, was the most prevalent missense mutation [63]. Then came the p.(E542K) mutation, which is caused by a single nucleotide alteration (c.1624 G > A) and causes an AA substitution of a K for an E at position 542. These two mutations are in the helical domain of *PIK3CA*. In the kinase domain, the most common mutation was p.(H1047R), which results from a single nucleotide change (c.3140A > G), leading to the substitution of histidine (H) at position 1047 with arginine (R) [63]. In HNSCC, *PIK3CA* messenger RNA (mRNA) overexpression is a common occurrence. Mutations and copy number gains (0.5–11%) are also seen in several PI3K regulatory subunits and other PI3K isoforms (PIK3CB, PIK3CD, and PIK3CG) [64].

Hyperactivity of PI3K plays a significant role in the development and progression of many cancers [65]. The hyperactivity of PI3K promotes the production of VEGF, which stimulates normal and tumor angiogenesis [66]. By phosphorylating Ser1177, activated AKT causes eNOS distribution in the vascular endothelium, which in turn leads blood vessels to produce nitric oxide (NO). This phenomenon aids in the regulation of vascular processes like angiogenesis, vascular remodeling, and vasodilation [67]. Moreover, significant quantities of HIF-1α are expressed when AKT activation occurs. HIF-1α is a crucial regulator of angiogenesis that can increase the expression of VEGF and other angiogenic factors, thus fostering angiogenesis. HIF-1α could encourage endothelial cell migration and proliferation and enhance vascular permeability, plasma protein exosmosis, and cellulose scaffold formation to aid in endothelial cell migration and offer support for vascular growth. HIF-1α could also induce the proteolytic enzyme system, which breaks down the extracellular matrix and encourages angiogenesis [68]. Angiogenesis plays an important role in the growth, metastasis, and lethality of tumors.

Hyperactivity of the PI3K and PI3K/AKT/mTOR signaling pathways, also through the following methods, plays a significant role in encouraging tumor invasion and metastasis. Activated AKT facilitates the invasion of tumors and increases Mdm2 expression. Mdm2 is the primary negative regulator of p53, promoting its degradation and thereby inactivating its tumor-suppressing function [69]. It also facilitates the spreading of tumors. It has been revealed that AKT1, a downstream PI3K molecule, and actin polarization promotion control the invasion and metastasis of breast cancer cells. Palladin is an actin-related protein that plays a key role in cell migration, controls the organization of the actin system, and contributes to the formation of the cytoskeleton. AKT1 can phosphorylate palladin’s Ser507, which controls the invasion and metastasis of cancer cells [70]. Cell invasion and metastasis are encouraged by the activation of matrix metalloproteinases (MMPs), a class of proteolytic enzymes that take part in the extracellular matrix’s degradation. MMP-2 and MMP-9 are two of the 23 different types of MMPs that are known to be essential for cell invasion. PI3K/AKT/mTOR can break down the extracellular matrix, increase the expression of MMP-2 at the mRNA and protein levels, and increase the invasion and metastasis of cancer cells [71].

### 2.2. AKT Mutations in HNSCC

Activating mutations or amplifications of AKT upstream factors, such as in *PTEN*, *PIK3CA*, growth factor, or cytokine receptors, typically cause changes in AKT activity by enhancing the expression and activity of one, two, or all three isoforms of AKT [72]. Genes encoding one of the three isoforms of the oncogenic protein AKT—which is known to be involved in controlling cell survival, proliferation, growth, apoptosis, and glycogen metabolism [73]—have been found to harbor gain-of-function missense mutations and amplification [74].

The overall pooled prevalence of *AKT* mutations in head and neck cancer was 2% [63]. The most frequent mutation was found in the hotspot p.(E17K), which is caused by a single nucleotide change (c.49 G > A) and leads to the AA substitution of a K for an E at position 17 [63].

### 2.3. MTOR Mutations in HNSCC

To start cell division, mTOR encourages cyclin D1 to attach to cyclin-dependent kinase (CDK). Elevated expression of cyclin D1 can abbreviate the cell cycle, hasten the development of cancer, and cause the cell cycle to move from the G1 to the S phase. Furthermore, mTOR controls the production of biological macromolecules, including lipids, proteins, and nucleotides, which supply the building blocks needed for the proliferation of cancer cells [71].

According to the meta-analysis published in 2021, the overall pooled prevalence of *MTOR* mutations in head and neck cancer was 3% [63]. The population with the highest frequency of *MTOR* mutations was Asia (4.1%), Europe (2.3%), and North America (1.7%). Missense mutations were more frequent, but none of the mutations have been reported more than once [63].

### 2.4. PTEN Mutations in HNSCC

The tumor suppressor PTEN plays a major role in PI3K/PIP3 signal termination by dephosphorylating PIP3 and converting it back to PIP2. Consequently, PTEN functions as a crucial negative regulator of the PAM pathway, which influences cell growth and survival; on the other hand, PTEN absence releases the inhibition of this intracellular signaling [75]. In both primary and metastatic breast cancer, *PTEN* loss typically results in hyperactivation of the PAM pathway, which in turn promotes cell proliferation [76,77]. Resistance to anticancer treatment is associated with both PTEN downregulation and PAM pathway activation [78].

**Figure 2:**
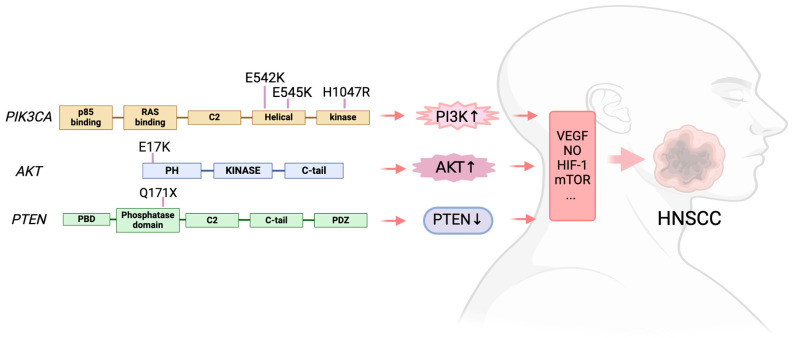
The PAM signaling mutations in HNSCC.

Numerous malignancies include mutations that cause *PTEN* to lose its function [79,80]. The prevalence of the *PTEN* mutation in head and neck cancer was reported at 4% by a meta-analysis including 4785 samples described in 57 articles [63]. According to this meta-analysis, forty percent of *PTEN* mutations are missense and 11% are nonsense mutations. In head and neck cancer, the most frequent mutations were p.(Q171X) and the single nucleotide alteration c.511C > T [63] (Figure 2).

## 3. Targeting the PAM Axis in Head and Neck Cancer

Recent characterization of molecular alterations in HNSCC has revealed that the PI3K/mTOR signaling pathway is the most frequently dysregulated pathway in this type of cancer [81]. Consequently, using targeted molecular therapies to reduce mTOR pathway activity could have anticancer effects in HNSCC.

### 3.1. PI3K Inhibitors

PI3K is a prominent pharmacological target for cancer treatment along the PAM pathway because of its remarkable correlation between hyperactivity and the evolution of human tumors, the production of boosted tumor microvessels, and a rise in the number of invasive cancer cells. Even though the Food and Drug Administration (FDA) has authorized several inhibitors, concerns about toxicity, sensitivity indicators, and resistance development persist [82]. It is noteworthy that PI3K inhibitors may be divided into three primary categories: pan-PI3K inhibitors (pan-PI3Ki), isoform-specific PI3K inhibitors (IS PI3Ki), and dual PI3K/mTOR inhibitors (dual PI3K/mTORi) [83]. All PI3K class I isoforms’ catalytic activity is suppressed by pan-PI3Ki. Therefore, regardless of the kind of *PI3K* gene or *PTEN* changes involved, these medications are often beneficial in cancers that produce large levels of PIP3.

Pan-PI3Ki may have a wider spectrum of action since it includes several molecular targets, but there is a growing danger of both on- and off-target toxicity [84]. Numerous pan-PI3Ki have been studied in clinical settings. Buparlisib (BKM120) is an orally bioavailable pan-PI3K inhibitor [84]. As a potent pan-class I PI3K inhibitor, buparlisib targets the ATP binding site of the p110 kinase domain. Its inhibitory effectiveness is significantly reduced against class IB p110γ but equally effective on class IA isoforms of p110α, β, and δ [85]. When taken orally, buparlisib is quickly absorbed, and the amount in the serum rises in direct proportion to the dosage [86]. Preclinical findings further suggested that its anticancer efficacy was due to an antiangiogenic impact [85] and the reduction of microtubular dynamics [87]. In cell lines with wild-type *PIK3CA* as well as mutant forms carrying any hotspot mutation of E542K, E545K, or H1047R, buparlisib decreased PI3K activity in vivo [88]. The most frequent adverse effects in a phase I dose-escalation trial for advanced solid tumors were tiredness, rash, altered liver function, and changes in glucose metabolism [89]. Results of the phase III trial about buparlisib in combination with chemotherapy (paclitaxel) compared to paclitaxel alone in patients with recurrent or metastatic head and neck squamous cell carcinoma are awaited (NCT04338399) (Table 1). Another pan-PI3Ki, PX-866, binds to Lys in the ATP catalytic site to irreversibly inhibit class I PI3K [90]. In xenograft models of human HNSCC, PX-866 prevented the growth of tumors. Two of these cases included *PIK3CA* gene amplification, while the other involved E545K [91]. Nevertheless, PX-866’s clinical studies did not yield encouraging outcomes [92]. Copanlisib, another pan-PI3Ki, combined with cetuximab demonstrated limited efficacy and unfavorable toxicity in HNSCC patients (NCT02822482) [93].

A more favorable side effect profile is possible with IS PI3Ki, a selective inhibitor of PI3Kα that has anticancer efficacy without affecting other isoforms of PI3K. Just three IS PI3Ki—alpelisib, duvelisib, and idelalisib—have received FDA approval. Alpelisib is a specific inhibitor of PI3Kα, which is the product of frequently mutated *PIK3CA* [94]. Alpelisib inhibits wild-type PI3Kα and PI3Kα with common *PI3KCA* mutations, such as E545K or H1047R, more potently than PI3Kδ or PI3Kγ [95]. Preclinical evidence also revealed that cancer cells with *PIK3CA* mutations are more susceptible to alpelisib’s PI3K inhibition. Reliable preclinical models are crucial for studying relevant molecular mechanisms and selecting the final interventions for clinical research. These models include immortalized-cell and primary tumor cultures in monolayer or 3D and animal models [96]. Preclinical models of HNSCC, which can be used to analyze the biological roles of genetic variants and altered gene expression, will be highly valuable for translating molecular discoveries into enhanced clinical care [97]. An extensive panel of cancer cell lines was used for an in vitro pharmacologic sensitivity screen, and the results showed that sensitivity to alpelisib was positively correlated with the presence of the *PIK3CA* mutation, amplification, or copy number increase. This finding was further supported by an in vivo investigation employing mouse models [95]. HNSCC cell lines with the *PIK3CA* H1047R mutation were more vulnerable to the antiproliferative impact of alepelisib than were cell lines with the wild-type *PIK3CA* [98]. The *PIK3CA* mutation was the best predictive factor that corresponded with a good response to alpelisib in another in vivo study, regardless of the location where it was found [95]. PI3K inhibitors are being investigated in conjunction with other targeted medicines since compensatory hyperactivation of *PIK3CA* is one of the main reasons for treatment resistance. As for duvelisib, several clinical trials are underway to test it for the treatment of advanced HNSCC (NCT05057247 and NCT04193293) (Table 1). Idelalisib, also known as CAL-101, has been approved by the FDA as the first-in-class PI3K inhibitor for hematological cancer treatment [99], but the possibility of using idelalisib in HNSCC remains unestablished. Currently, there are no clinical trials exploring the efficacy and risks of Idelalisib for head and neck cancer.

### 3.2. AKT Inhibitor

MK-2206, GDC0068, and D-21266 are the three AKT inhibitors that have been employed in HNSCC studies.

MK-2206 is an orally active, allosteric inhibitor of AKT that prohibits membrane recruitment and phosphorylation of AKT [100]. MK-2206 treatment is adequate to prevent HNSCC migration and chemotaxis in vitro. Treatment with MK-2206 improves survival in an orthotopic model by reducing the size of the main tumor and cervical metastases [101]. However, the phase II clinical trials of MK-2206 in HNSCC were terminated due to the limited activity observed [102].

Ipatasertib (GDC0068) is an orally administered, ATP-competitive, highly selective inhibitor of AKT [103]. It suppresses downstream signaling and raises AKT phosphorylation. Ipatasertib is now being evaluated alone (NCT05172258) or in combination with chemotherapy (NCT05172245) for the treatment of HNSCC in phase I or II trials.

Perifelosine (D-21266) is an oral alkylphospholipid that prevents AKT phosphorylation [104]. Preclinical research using a rat mammary cancer model revealed that perifosine had antitumor properties [105]. Phase I trials of perifosine show that when administered in accordance with a loading and maintenance schedule, perifosine can safely maintain drug concentrations that are close to those attained in preclinical models, showing indications of antitumor activity [106]. The toxicities observed included gastrointestinal toxicity, arthralgia, and fatigue. The phase II trial of perifosine in HNSCC patients reveals that the doses and schedule used lack single-agent activity [104].

### 3.3. mTOR Inhibitor

The first-generation mTOR inhibitor rapamycin (sirolimus) has been extensively studied and is known to form a complex with FKBP12, inhibiting the activity of mTORC1. In the context of head and neck cancer (HNC), several Phase I and II trials have evaluated the safety and efficacy of rapamycin in patients with stage II-IVA HNSCC. For instance, in a trial involving 16 patients, rapamycin was well tolerated, with 25% of patients showing RECIST responses, and downstream proteins such as AKT, S6, 4EBP, and Ki67 exhibited promising effects (NCT01195922) [107]. However, another trial involving four HNC patients, despite the combination regimen with bevacizumab, showed no objective responses by RECIST criteria, although there was a reduction in tumor size [108]. Overall, while some studies did not show objective responses, the long-term use of mTOR inhibitors like rapamycin, especially in combination regimens, demonstrated tolerability and potential efficacy in HNC patients, suggesting promising effects with careful adverse effects management [109].

The second-generation mTOR inhibitor everolimus (EVE), particularly everolimus (EVE), has been evaluated through a comprehensive review of several Phase I and II clinical trials [109,110,111,112,113] encompassing patients with stage two to four HNC. Of these trials, most were completed successfully, while two were terminated due to adverse effects when combined with other therapies (NCT01009346, NCT01057277) [109].

Among the completed trials, two did not demonstrate significant benefits. For instance, in the NCT01051791 Phase II study, where EVE was administered to recurrent/metastatic HNC patients, adverse effects led to discontinuation in 33% of patients, and the results failed to improve clinical benefits, with no objective responses observed and a clinical benefit rate (CBR) of 28%, along with median progression-free survival (PFS) and overall survival (OS) of 1.5 and 4.5 months, respectively [112].

Similarly, in the NCT00942734 Phase II study, the addition of EVE to the erlotinib regimen did not show significant benefits in 35 platinum-resistant/metastatic HNC patients, despite tolerable adverse effects. The results indicated a median PFS of 11.9 weeks and a median OS of 10.25 months [110]. However, four trials did show clinical benefits. For example, in the NCT01283334 Phase I + II study, EVE combined with cetuximab and carboplatin yielded encouraging results, with a 61.5% objective response rate (all partial responses) and a PFS of 8.15 months [111]. The NCT01333085 Phase I + II Study, using the CAPRA regimen (everolimus, carboplatin, and paclitaxel) as induction therapy, showed promising results in 50 locally advanced HNC patients, with a 79% overall response rate and encouraging translational results indicating a direct effect of EVE in tumors. Furthermore, the NCT00935961 Phase I study using EVE combined with cisplatin and docetaxel in advanced HNC patients demonstrated a progression-free survival rate of 87.5% at 1 year and 76.6% at 2 years [113]. Similarly, the NCT00858663 Phase I study employing EVE with cisplatin and radiotherapy in stage 2-4 HNC patients showed a two-year progression-free survival of 85% and a two-year overall survival of 92% [114].

Despite the two trials that did not yield significant beneficial results, whether using EVE as monotherapy or in combination with erlotinib, the potential of EVE as part of combined therapy in HNC remains promising. The positive outcomes observed in trials combining EVE with various agents, such as cetuximab, carboplatin, paclitaxel, docetaxel, and radiotherapy, indicate the potential for EVE as a component of combined therapy options in HNC, although further investigation is warranted to delineate optimal treatment strategies.

### 3.4. The PAM Pathway Inhibitor Resistance

**Figure 3:**
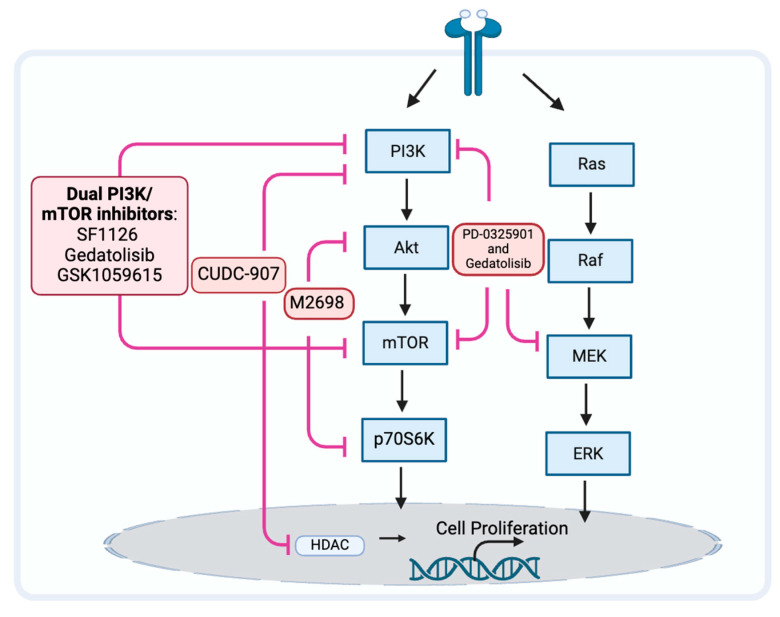
Strategy may prevent tumor resistance to PAM pathway inhibitors.

Because the PAM pathway may not be fully inhibited, may reactivate, or other prosurvival pathways may be activated, the PAM pathway inhibitors may be ineffective [115,116]. When a single node in the PAM pathway, such as mTOR, is inhibited, a negative feedback loop can be released, which often activates AKT in response and ends up with a compensatory activation [116]. When inhibiting downstream signaling molecules of PAM, such as mTOR, simultaneous inhibition of upstream signaling molecules may help avoid compensatory activation and resistance (Figure 3). When compared to mTOR blockade alone, inhibition of both PI3K and mTOR may improve antitumor effectiveness [117,118]. The drugs SF1126, dactolisib (NVP-BEZ235), voxtalisib (XL765), gedatolisib (PKI-587), and GSK1059615 are examples of dual PI3K/mTOR inhibitors (PI3K/mTORi) [65,119]. It is currently unclear what effect SF1126, dactolisib, and voxtalisib have on head and neck cancer. Relevant clinical studies on SF1126 have been completed, and the results are being organized (NCT0264412). Gedatolisib is currently being used in phase I clinical trials exploring the efficacy for HNSCC (NCT03065062). GSK1059615 was reported to induce antitumor activity, possibly by provoking the programmed necrosis pathway in vitro and in a mouse model [120].

M2698 is an oral dual inhibitor of p70S6K and AKT, which blocks p70S6K to inhibit the PAM pathway while simultaneously targeting AKT to overcome the compensatory feedback loop [121]. A clinical investigation including patients with advanced cancer [122] and mouse models [121] indicated this potential effect of p70S6K/AKT dual inhibition. Currently, there are no reported studies on the use of this dual inhibitor for HNSCC.

Modulation of the PAM pathway signaling or contemporaneous changes in other pathways that activate parallel tumor signaling pathways can activate compensatory prosurvival programs and result in PAM pathway inhibitor resistance [122,123]. By concurrently inhibiting alternative pathways, cancer cells that were previously susceptible to PI3K inhibition can now evade treatment resistance by downregulating other signaling proteins.

There is a known connection between the PI3K/AKT/mTOR and RAS/RAF/MEK/ERK pathways at several nodes, and this interaction may result in a potential pathway convergence for the development of therapeutic combinations [124]. MEK inhibitor PD-0325901 overcomes resistance to PI3K/mTOR inhibitor gedatolisib (PF-05212384) and potentiates antitumor effects in HNSCC cell lines [125]. Further research is needed to confirm the effects and side effects for patients with HNSCC.

The epidermal growth factor receptor (EGFR) family is a subclass of RTK proteins and consists of four members: EGFR (ErbB1, HER1), ErbB2 (HER2), ErbB3 (HER3), and ErbB4 (HER4). Patients with cancer that overexpresses HER2 now have a considerably better prognosis thanks to anti-HER2 treatments. Constitutive stimulation of the PI3K pathway is one mechanism of resistance to anti-HER2 treatments. The therapeutic drawbacks of single-agent anti-HER2 treatment may be addressed by combination therapy, including HER2 and PAM inhibitors [126].

Histone deacetylases (HDACs) are epigenetic modifiers that play crucial roles in many key biological functions. HDAC inhibitors like CUDC-907, which inhibit HDAC and the PI3K pathway, are a relatively new emerging class of anticancer drugs [127]. Concurrent inhibition of HDAC can also downregulate other signaling proteins and bypass treatment resistance in cancer cells that are resistant to PI3K inhibition due to alternative pathway activation. The simultaneous targeted inhibition of HDACs and PI3Ks with their respective inhibitors in combination showed synergistic therapeutic efficacy. An in vitro discovery showing that the administration of an HDAC inhibitor effectively overcomes mTOR inhibitor resistance in lymphoma cells provided evidence for the potential advantages of dual inhibition [128]. Additionally, combined inhibition of PI3K and HDAC reverses platinum drug resistance by preventing DNA repair and multidrug resistance transporters, according to an in vivo investigation [129]. Combination inhibition of HDAC with dual PI3K-mTOR inhibitors possesses an antitumor effect against HNSCC in vivo [130]. The evaluation of the benefits and side effects for patients with HNSCC requires additional investigation.

The combination of PAM inhibitors reduces the occurrence of resistance and holds greater potential for treating HNSCC. However, it is crucial to determine the optimal combination for each patient. Personalized preclinical platforms to test gene expression profiles and drug sensitivity of patient samples may help in selecting the appropriate combination. Additionally, special attention must be given to managing the toxicity of innovative combination approaches.

## 4. Conclusions

Targeting the PAM signaling pathways as a precision therapeutic approach in HNSCC has been extensively studied in experimental models and clinical trials, as the significance of the PAM signaling pathways in HNSCC has been well-documented. Moreover, there is now a justification for combining the PAM with different routes, targeting itself and alternative pathways to increase their antitumor efficaciousness. Overall, we anticipate that the creation of innovative PAM co-targeting techniques may result in long-lasting effects and cancer remission, extending the survival and improving the quality of life of HNSCC patients.

## Figures and Tables

**Table 1:** Clinical trials targeting the PAM signaling pathway in head and neck cancer.

Classification	Drug	Trial ID	Patient	HNSCC Types	Phase	Intervention	Status
Pan-PI3Ki	Buparlisib (BKM120)	NCT01816984	R/M HNSCC	Unspecified	I/II	BKM120 and cetuximab	Completed
		NCT02113878	LA HNSCC	Unspecified	Ib	BKM120 with cisplatin and XRT	Completed
		NCT04338399	R HNSCC	Unspecified	III	Buparlisib and paclitaxel	Recruiting
	PX-866	NCT01204099	LA/R/M HNSCC	Unspecified	I/II	PX-866 and docetaxel	Completed, awaiting results
		NCT01252628	P//R/M HNSCC	Unspecified	I/II	PX-866 and cetuximab	Completed, awaiting results
	Copanlisib (BAY80-6946)	NCT02822482	R/M HNSCC	Oral cavity, oropharynx, larynx, or hypopharynx	II	Copanlisib and cetuximab	Completed
IS PI3Ki	Alpelisib (BYL719)	NCT03292250	R/M HNSCC	Unspecified	II	BYL719	Completed, awaiting results
		NCT01602315	R/M HNSCC	Unspecified	Ib/II	BYL719 and cetuximab	Completed
		NCT02051751	R/M HNSCC	Unspecified	I	BYL719 and paclitaxel	Completed
		NCT02145312	R/M HNSCC	Unspecified	II	BYL719	Completed, awaiting results
	Duvelisib (VS-0145)	NCT04193293	R/M HNSCC	Oral cavity, oropharynx, hypopharynx, or larynx	I/II	Duvelisib and pembrolizumab	Completed, awaiting results
		NCT05057247	R/M HNSCC	Oral cavity, oropharynx, larynx, hypopharynx, nasal cavity, and the paranasal sinuses	II	Duvelisib and docetaxel	Active
dual PI3K/mTOR inhibitor	SF1126	NCT0264412	R/P HNSCC	Unspecified	II	SF1126	Completed, awaiting results
	Gedatolisib (PF-05212384)	NCT03065062	LA HNSCC	Unspecified	I	Gedatolisib and palbociclib	Recruiting
		NCT02069158	HNSCC	Unspecified	I	Gedatolisib with paclitaxel and carboplatin	Completed, awaiting results
AKT inhibitors	MK-2206	NCT01349933	R/M HNSCC	Nasopharyngeal	II	MK-2206	Completed
	Ipatasertib (GDC0068)	NCT05172258	R/M HNSCC	Oral cavity, oropharynx, hypopharynx, and larynx	II	Ipatasertib	Recruiting
		NCT05172245	LA HNSCC	Oropharynx, hypopharynx, larynx, oral cavity, nasal cavity, maxillary and other paranasal sinuses	I	Ipatasertib and chemotherapy	Recruiting
	Perifosine (D-21266)	NCT00062387	R/M HNSCC	Unspecified	II	Perifosine	Completed
mTOR inhibitor	rapamycin (sirolimus)	NCT01195922	LA HNSCC	Oral cavity or oropharynx	I/II	Rapamycin	Completed
		NCT01256385	R/M HNC	Lip, oral cavity, laryngeal	II	Temsirolimus and cetuximab	Completed
		NCT01015664	R/M HNSCC	Unspecified	I/II	Temsirolimus, cisplatin, and cetuximab	Completed, awaiting results
	RAD001 (everolimus)	NCT00858663	HNC	Oral cavity or oropharyngeal	I	Radiation, everolimus, and cisplatin	Completed, awaiting results
		NCT01313390	R/M HNC	Lip, oral cavity, larynx, hypopharynx, nasopharynx, paranasal sinus and nasal cavity	I/II	Everolimus and docetaxel	Completed, awaiting results
		NCT01051791	LA/R HNSCC	Unspecified	II	Everolimus	Completed
		NCT01133678	LA HNSCC	Hypopharynx, oral cavity, oropharynx, larynx	II	Everolimus and placebo	Completed
		NCT01111058	LA HNC	Lip, nasopharynx, nasal cavity, paranasal sinus, skin	II	Everolimus and placebo	Completed
		NCT01333085	LA HNSCC	Oral cavity, oropharynx, larynx, or hypopharynx	I/II	Everolimus, carboplatin, and paclitaxel	Completed, awaiting results
		NCT01283334	LA HNSCC	oral cavity, oropharynx and larynx, hypopharynx or paranasal sinus	I/II	Everolimus, carboplatin, and cetuximab	Completed
		NCT00942734	R HNSCC	Oropharyngeal and other	II	Everolimus and erlotinib	Completed
		NCT00935961	LA HNSCC	Unspecified	I	Everolimus, docetaxel, and cisplatin	Completed, awaiting results
		NCT01058408	LA HNSCC	oropharynx, hypopharynx, larynx primaries, nasopharynx	I	Everolimus, cisplatin, and radiation	Completed, awaiting results
		NCT03578432	LA HNSCC	Unspecified	I	Everolimus and radiation	Completed, awaiting results
		NCT01009346	R/M HNSCC	Unspecified	I/II	Everolimus, cetuximab, and cisplatin/carboplatin	Terminated
		NCT01057277	LA HNSCC	Oral cavity, oropharynx, hypopharynx, or larynx	I	Everolimus, cisplatin, and concurrent radiation	Terminated

pan-PI3Ki, pan-PI3K inhibitors; IS PI3Ki, isoform-specific PI3K inhibitors; R, recurrent; M, metastatic; LA, locally advanced; P, progressive.
